# Takayasu’s Arteritis With Acute Severe Aortic Regurgitation Requiring a Bio-Bentall Procedure

**DOI:** 10.7759/cureus.26969

**Published:** 2022-07-18

**Authors:** Polsha Jules, Oscar Valencia, Damian Valencia, Ananya Reddy, Rehan Ahmed

**Affiliations:** 1 Department of Internal Medicine, Kettering Health Network, Dayton, USA; 2 Department of Internal Medicine, Loyola University Chicago, Chicago, USA; 3 Department of Cardiovascular Medicine, Kettering Medical Center, Dayton, USA; 4 Public Health, Milken School of Public Health, George Washington University, Washington, USA; 5 Department of Cardiovascular Medicine, Kettering Health Network, Dayton, USA

**Keywords:** ischemic bowel, bio-bentall, thoracic aortic aneurysm, aortic regurgitation, aortitis, vasculitis, takayasu’s arteritis

## Abstract

Aortitis is a rare form of vasculitis that is associated with significant morbidity and mortality through the development of aneurysms, aortic rupture, dissection, and thrombotic occlusions. Common causes for non-infectious large vessel vasculitis include Takayasu's arteritis and giant cell arteritis. Delayed diagnosis and treatment can be devastating, resulting in lifelong disability or death. Here we present an unfortunate case of Takayasu's arteritis with aortitis and acute severe aortic regurgitation in a young patient requiring an emergent Bio-Bentall procedure and bowel resection.

## Introduction

Isolated aortitis is a rare form of vasculitis that is associated with significant morbidity and mortality through the development of unstable aneurysms, aortic rupture, dissection, and thrombotic luminal occlusions [1]. Aortitis can be infectious (related to tuberculosis, syphilis, salmonella, etc.) or noninfectious (related to connective tissue disorders and immunologic conditions) [2-3]. The most common causes of noninfectious large vessel vasculitis are Takayasu's arteritis (TA) and giant cell arteritis (GCA) [2]. Other less common etiologies include rheumatoid arthritis, systemic lupus erythematosus (SLE), Crohn's disease, anti-neutrophil cytoplasmic antibody (ANCA)-associated vasculitis, human leukocyte antigen B27 (HLA-B27)-associated spondyloarthropathies, Behçet's disease, Marfan's disease, Cogan syndrome, and sarcoidosis [2]. GCA is most common in older (mean age 75 years old) females of European ancestry, with an incidence of 18.8 per 100,000 in patients greater than 50 years old [4]. TA primarily affects younger (<40 years old) females of Asian and African ancestry, with an incidence of 2.6 per 1 million patients [5-6]. In regards to histopathological findings, both GCA and TA have significant overlap with the demonstration of inflammatory lymphocytic cellular infiltrate of the aortic media, adventitia, and vasa vasorum, granuloma formation, and the presence of multinucleated giant cells [7]. Delayed diagnosis and treatment can be devastating, resulting in lifelong disability or death. Here we present a case of fulminant Takayasu's aortitis in a young African female requiring an emergent Bio-Bentall procedure and bowel resection. 

## Case presentation

A 36-year-old African female with recent immigration from the Ivory Coast, Africa, presented with acute onset shortness of breath. The patient reported an associated severe exertional dyspnea, orthopnea, nausea, and vomiting. On initial evaluation, hemodynamics were stable (143/57 mmHg, 103 BPM, 24 respirations/minute, 99% SpO2) on 2 L nasal cannula supplemental oxygen. The patient was underweight (BMI 18.5 kg/m2) and appeared to be in respiratory distress. The examination was positive for bibasilar crackles, diminished breath sounds bilaterally, tachycardia with a loud grade IV diastolic murmur at the right upper sternal border, jugular venous distension, and cold extremities with weak pulses. 

**Figure 1:**
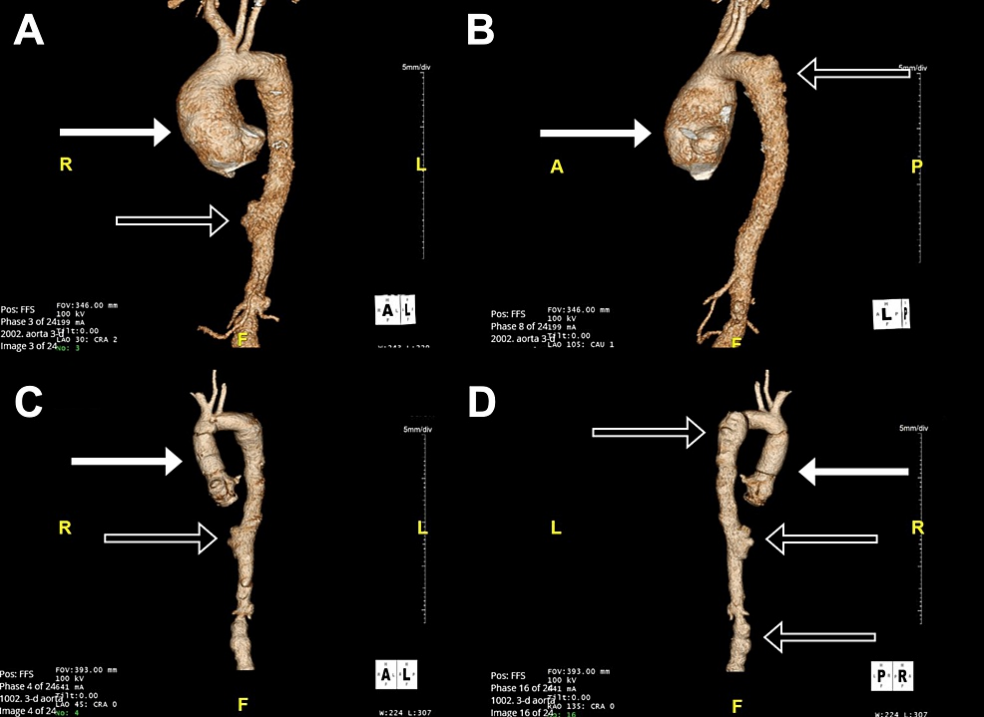
CT angiogram of the chest and abdomen with reconstructed 3D images of the aorta (A) Anterior-posterior 3D reconstructed image of the aorta showing the ascending aortic aneurysm (white arrow) measuring 5.9 cm at maximal diameter. A saccular aneurysm with serrated/irregular contour (black arrow) measuring 3.4 cm at maximal diameter is also present in the distal descending thoracic aorta. (B) Posterior-anterior 3D reconstructed image of the aorta again showing the ascending aortic aneurysm (white arrow) and eccentric dilation of the proximal descending aorta with serrated/irregular contour (black arrow) measuring 3.8 cm at maximal diameter. (C) Anterior-posterior 3D reconstructed image of the aorta showing aortic root graft (white arrow) post-Bio-Bentall procedure. The saccular aneurysm in the distal descending aorta is again visualized (black arrow). (D) Posterior-anterior 3D reconstructed image of the aorta showing aortic root graft (white arrow) post-Bio-Bentall procedure. Multiple saccular aneurysms can be seen in the proximal descending aorta, distal descending aorta, and infrarenal abdominal aorta (black arrows).

**Figure 2:**
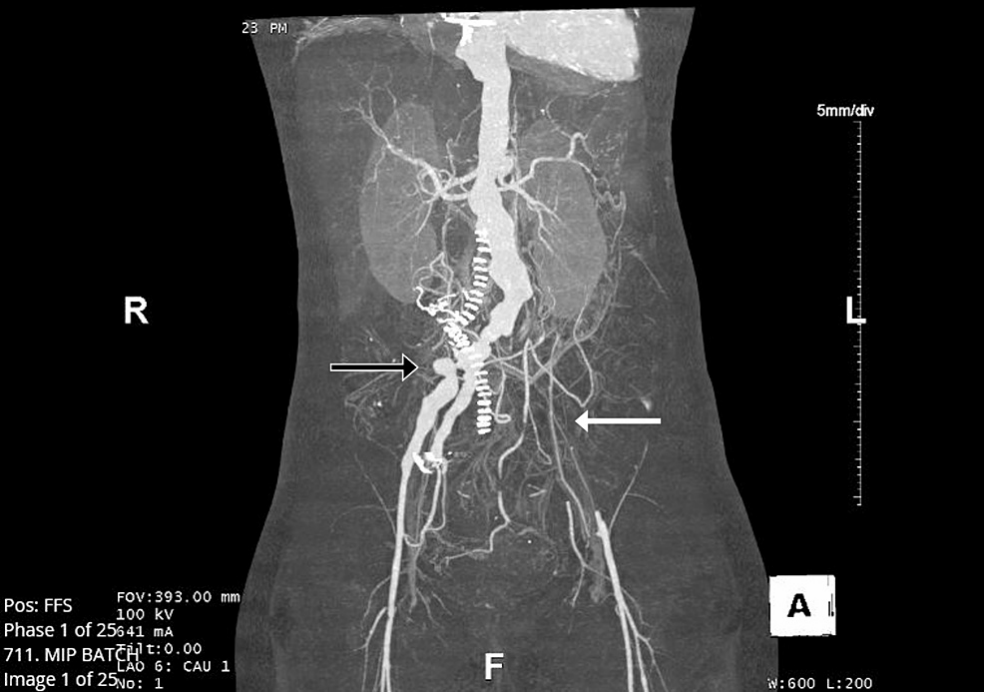
CT angiogram of the abdomen and pelvis Angiogram of the abdomen and pelvis showing a saccular aneurysm at the bifurcation of the right common iliac artery (black arrow) with focal high-grade stenosis at the right external iliac artery and proximal occlusion of the right superficial femoral artery. An occlusive thrombus is visualized in the left common iliac artery, left internal iliac artery, and left external iliac artery (white arrow).

**Figure 3:**
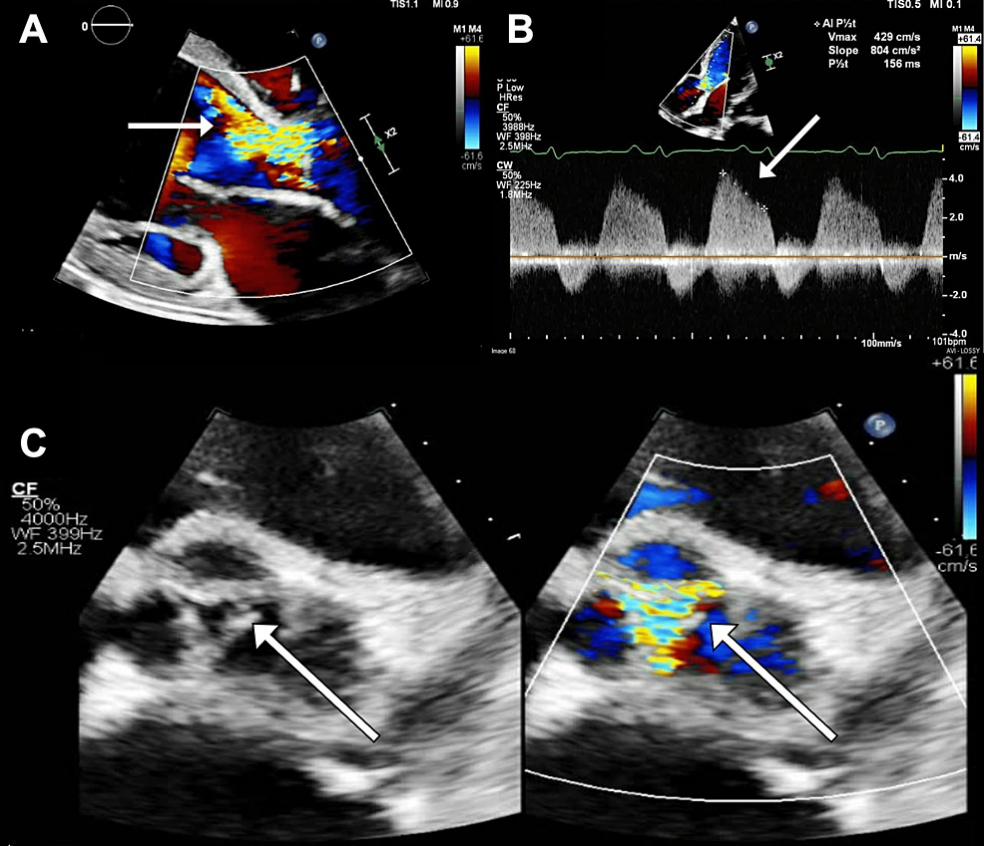
2D transthoracic echocardiography (A) 2D transthoracic echocardiography (TTE) parasternal long axis zoom in view with color Doppler of the aortic valve showing severe regurgitation. (B) 2D-TTE apical five-chamber zoom in view with color Doppler of the aortic valve showing severe regurgitation by continuous wave Doppler assessment (pressure half time of 156 milliseconds). (C) 2D-TTE short axis zoom in view of the aortic valve with and without color Doppler showing severe regurgitation during diastole.

Brain natriuretic peptide (BNP) was significantly elevated (2,688 pg/ml). The electrocardiogram (ECG) showed sinus tachycardia with deep T-wave inversion in the inferior leads. A chest roentgenogram (CXR) revealed bilateral pulmonary edema. Computed tomography angiography (CTA) of the chest and abdomen showed an ascending thoracic aortic aneurysm (maximal diameter 5.9 cm) and an eccentric dilation of the proximal descending aorta (maximal diameter 3.8 cm) with serrated/irregular contour (Figure 1A, 1B). Multiple saccular aneurysms were noted in the distal thoracic aorta (Figure 1A), infrarenal abdominal aorta, and right iliac artery (Figure 1C, 1D). There was a focal high-grade stenosis of the right external iliac with partial thrombosis of a saccular aneurysm at the common iliac artery bifurcation (Figure 2). A proximal occlusion of the right superficial femoral artery was also present. There was an occlusive thrombus in the left common iliac artery, left internal iliac artery, and left external iliac artery (Figure 2). A high-grade stenosis of the proximal splenic artery, total occlusion of the superior mesenteric, and inferior mesenteric arteries were also noted. 2D transthoracic echocardiography (TTE) revealed a markedly dilated left ventricle with an ejection fraction of 20% and grade III diastolic dysfunction. The ascending aortic aneurysm was also seen with associated severe wide open aortic regurgitation (Figure 3A, 3B, 3C). 

The patient was taken for emergent surgical intervention for acute severe aortic regurgitation. A Bio-Bentall Procedure was performed with resection of the ascending aortic aneurysm and aortic valve. The aortic valve was replaced with a 25 mm Freestyle tissue valve (Medtronic, Dublin, Ireland), and the ascending aorta was repaired with a 26 mm Gelweave graft (Terumo Aortic, Inchinnan, United Kingdom). 

Pathological specimens of the aortic valve showed mild myxoid degenerative changes. Aortic tissue was found to have focal mononuclear infiltrates with scattered inflammatory infiltrates at various locations, including the intimal surface and around small caliber branching vessels. The inflammatory infiltrate included neutrophils, histiocytes, lymphocytes, and plasma cells. Immunohistochemical stains show the lymphocytes to be a mixture of CD3+ T-cells and CD79a+ B-cells. CD68 was positive on histiocytes. Elastic stain showed disruption. Special stains for acid-fast and fungal organisms were negative. No definitive bacteria was identified on the gram stain. The overall histologic findings were unusual and concerning for non-infectious granulomatous aortitis. 

Due to severe abdominal pain postoperatively, a repeat scan of the abdomen was obtained, which showed changes consistent with extensive ischemic bowel. The patent was taken back to the operating room for exploratory laparotomy. Resection of the bowel was performed from the ligament of Treitz to the ascending colon with primary anastomosis from the proximal jejunum to the mid-transverse colon. In consultation with a tertiary care center, the decision was made to defer corticosteroid therapy for granulomatous aortitis due to concerns over bowel surgical anastomosis deterioration. On postoperative day six, the patient again developed severe abdominal pain, prompting repeat imaging. A repeat CT scan of the abdomen showed an enlarging descending abdominal aortic aneurysm with pneumatosis and changes consistent with recurrent bowel ischemia. At this point, serologic profiles (anti-neutrophil cytoplasmic antibody, rheumatoid factor, cyclic citrullinated peptide, anti-nuclear antibody) obtained early in the hospital course returned negative. After discussion with the patient, it was decided to transfer the patient to a tertiary care center for repeat surgical exploration with possible bowel transplantation in the near future.

## Discussion

Thoracic aortic aneurysm (TAA) represents approximately one-third of admissions involving aortic aneurysms [8]. Compared with degenerative or genetic connective tissue anomalies, aortitis is a much less common cause of TAA. In the case detailed above, the patient had no evidence of infection, no smoking history, trauma, genetic, or systemic disease prior to admission, which would predispose her to aortic pathologies. Despite the patient's relatively good health, she developed a large ascending TAA complicated by severe wide open aortic regurgitation and heart failure. The diagnosis of TAA-related non-infectious aortitis is uncommon, only accounting for 2.8% of TAAs, and is most commonly done with histopathology following an aortic aneurysm repair [9]. The rate for TAA dissection or rupture is approximately 2% per year for aneurysms less than 5 cm in diameter, 3% per year for aneurysms 5.0-5.9 cm, and 7% per year for those 6.0 cm or larger [10]. In the case presented, the resected portion of the TAA showed inflammatory infiltrate, along with patchy adventitial and intimal fibrosis, consistent with granulomatous aortitis associated with Takayasu's arteritis [2]. The lack of rheumatoid nodules or medial inflammation with necrosis argue against other causes of non-infectious aortitis (i.e., rheumatoid arthritis and GCA) [2]. Due to significant overlap in regards to histopathological features, diagnosis must take into account clinical features as well [10]. The likely diagnosis in the case presented is Takayasu's arteritis, given the patient's young age, ancestral origin, and histopathological findings. Given the distribution of the disease, the patient would classify as type V Takayasu's arteritis with distal thrombotic sequelae in the lower extremities. 

There are five types of TA categorized by the vessel segments affected (ascending aorta, aortic arch, aortic arch branches, descending thoracic aorta, abdominal aorta, and renal arteries) [11]. See Table 1 for a description of each TA type [11]. If the coronary or pulmonary arteries are involved, a distinction is added to the type, C(+) or P(+), respectively. A small European study reports type I as the most common variant (25.6%), followed by type V (20.9%), type IIa (18.6%), type IV (13.9%), type IIb (11.7%), and type III (9.3%) [12].

**Table 1:** Characterization of Takayasu's arteritis by vessel involvement

Type	Vessel involvement
Type I	Branches from the aortic arch (brachiocephalic trunk, left common carotid artery, left subclavian artery)
Type IIa	Ascending aorta, aortic arch and its branches
Type IIb	Ascending aorta, aortic arch and its branches, thoracic descending aorta
Type III	Thoracic descending aorta, abdominal aorta, and/or renal arteries
Type IV	Abdominal aorta and/or renal arteries
Type V	Combined features of types IIb and IV

Initial treatment strategies for TA should include a combination of high-dose Prednisone (initial dose of 0.5-1 mg/kg/day) and glucocorticoid-sparing agents (typically Methotrexate 15-25 mg/week or Azathioprine 1-2 mg/kg/day) [13-14]. See Table 2 for initial medical therapies and dosing [13-14]. In cases of large aneurysms, dissections, or vessel thrombosis, open surgical repair or endovascular percutaneous repair should be considered. Emerging studies are currently evaluating the role of plasmapheresis, leukocytapheresis, and lymphocytoplasmapheresis in the treatment of TA [15].

**Table 2:** Initial treatment strategies for Takayasu's arteritis

Glucocorticoid agents	Glucocorticoid sparing non-biologic agents	Glucocorticoid sparing biologic agents
Prednisone 0.5-1 mg/kg/day	Methotrexate 15-25 mg/week	Etanercept 25 mg twice weekly
(maximum daily dose of 60-80 mg)	Azathioprine 2 mg/kg/day	Infliximab 3-5 mg/kg
Methylprednisolone 500–1,000 mg/day	Cyclophosphamide 50-100 mg/day	
	Mycophenolate mofetil 1.5-3 g/day	
	Leflunomide 20 mg/day	
	Cyclosporin 3 mg/kg/day	

## Conclusions

Aortitis is a rare form of large vessel vasculitis that is associated with significant morbidity and mortality. The presenting symptoms can be nonspecific, making diagnosis difficult. This case displays the severity of complications that can arise. Our patient was ultimately diagnosed with Takayasu’s arteritis with an associated large thoracic aortic aneurysm resulting in severe acute aortic regurgitation requiring a Bio-Bentall procedure.
